# Unveiling the silent threat of new onset atrial fibrillation in covid-19 hospitalized patients: A retrospective cohort study

**DOI:** 10.1371/journal.pone.0291829

**Published:** 2024-01-19

**Authors:** Muhammad Shakir, Syed Muhammad Hassan, Ursala Adil, Syed Muhammad Aqeel Abidi, Syed Ahsan Ali

**Affiliations:** 1 Department of Surgery, The Aga Khan University Hospital Karachi, Karachi, Pakistan; 2 Department of Internal Medicine, The Aga Khan University Hospital Karachi, Karachi, Pakistan; 3 Department of Family Medicine, The Aga Khan University Hospital Karachi, Karachi, Pakistan; 4 The Aga Khan University Medical College Karachi, Karachi, Pakistan; Hamdard University Hospital, PAKISTAN

## Abstract

**Background:**

COVID-19, a highly infectious respiratory disease, has been associated with a range of cardiovascular complications. One of the most commonly reported cardiovascular issues in COVID-19 patients is the development of arrhythmias. Among all types of arrhythmias, atrial fibrillation is the most frequently observed. Atrial fibrillation is characterized by an irregular and often rapid heartbeat, and it can be a serious and potentially life-threatening condition.

**Objective:**

To investigate the incidence and association of new onset atrial fibrillation in COVID-19 hospitalized patients and its impact on survival.

**Method:**

A retrospective cross-sectional study that encompassed all patients, both positive and negative for COVID-19, who were consecutively admitted to the Aga Khan University Hospital in Karachi, a tertiary care facility, from June 2021 to December 2021.

**Results:**

A total of 1,313 patients who met the inclusion criteria of our study were enrolled as participants. These patients were then stratified into two groups based on COVID-19 status: the study group (COVID-19 positive) comprised 626 (47.7%) patients and the control group (COVID-19 negative) consisted of 687 (52.3%) patients. The incidence of new-onset atrial fibrillation was 85 (13.6%) in COVID-19 positive compared to 43 (5.2%) in COVID-19 negative group. The study found a strong association between COVID-19 and new-onset atrial fibrillation in both univariate (unadjusted odd ratio 2.35 [95% CI, 1.60–3.45], p-value < 0.01) and a multiple-adjusted regression analysis (adjusted odd ratio 3.86 [95% CI, 2.31–6.44], p-value < 0.01).

**Conclusion:**

These findings highlight the importance of vigilant monitoring of cardiovascular complications in COVID-19 patients, especially those with pre-existing conditions that predispose them to the development of atrial fibrillation. The study underscores the need for prompt recognition and management of new onset atrial fibrillation in COVID-19 patients, as this may mitigate the risk of adverse outcomes and improve overall prognosis.

## Introduction

Atrial fibrillation (AF) is a common dysrhythmia characterized by chaotic heart rhythm and irregular ventricular rate [1]. It occurs when electrical impulses fire from multiple sites in both atria, overwhelming the ventricles and causing abnormal blood pumping. AF also increases the risk of thromboembolic events, particularly stroke, due to blood stasis [2]. The Framingham Heart study reports a lifetime risk of AF in older adults as 1 in 5 [3].

Patients who are critically ill or admitted in ICU with sepsis or shock like condition are more susceptible to develop new onset atrial fibrillation (NOAF) [4]. Incidence varies substantially amongst critically ill and ICU patients; however, studies demonstrate it from 1.7% to 43.9%. NOAF is usually thought to be a consequence of critical illness, including inflammation, electrolyte disturbances, or pro arrhythmic medications, namely vasopressors and inotropes [5, 6].

Coronavirus disease 2019 (COVID-19) emerged in early December 2019 in Wuhan (Hubei, China) as a viral infection with pneumonia and respiratory syndrome caused by a new coronavirus, severe acute respiratory syndrome coronavirus 2 (SARS-CoV-2). The spectrum of infection ranges from asymptomatic to critical. Among hospitalized patients, the proportion of critical disease is higher, with 30% of patients requiring intensive care. Most of the fatal cases occurred in patients with advanced age or underlying medical comorbidities such as cardiovascular disease, arterial hypertension, diabetes mellitus, chronic lung disease, chronic kidney disease, obesity, or cancer [7]. Due to its rapid world-wide, human to human transmission via droplets and direct contact, it has been declared as pandemic by the World Health Organization [WHO] in March 2020 [8]. The mortality rate is higher in COVID infected elderly population particularly those who have underlying comorbid conditions [10].

Arrhythmias are often reported in COVID-19 patients, with AF being the most common form [8, 9] A systematic review of 21,653 hospitalized COVID-19 patients demonstrated a pooled prevalence of atrial fibrillation (AF) of 11%, with a notably higher prevalence among older patients (≥60 years) compared to younger patients (<60 years) (13% vs. 5%). Geographically, Europeans exhibited the highest AF prevalence at 15%, followed by Americans (11%), Asians (6%), and Africans (2%). Moreover, the prevalence of AF was six times greater in patients with severe COVID-19 compared to those with non-severe COVID-19 (19% vs. 3%) [10]. In another study involving a cohort of 30,999 COVID-19 hospitalized patients from 120 institutions across the United States, 5.4% experienced new-onset AF during their index hospitalization [11]. Additionally, an independent study involving 187,716 COVID-19 patients, the observed prevalence of AF was up to 8% (95% CI: 6.3–10.2%, 95% prediction intervals (PI): 2.0–27.1%) within the affected population [12]. New-onset AF in COVID-19 patients is a poor prognostic predictor and associated with poor outcomes as it is associated with higher incidence of thromboembolic events, bleeding risk and longer hospital stays [13, 14].

Infections and inflammation promote AF through various mechanisms. They trigger an immune response, releasing inflammatory mediators and cytokines that directly affect the electrical properties of the heart, disrupting its normal rhythm. This can lead to electrical remodeling and fibrosis in the atrial tissue, causing conduction abnormalities and facilitating AF. Additionally, infections and inflammation can disrupt the balance between the sympathetic and parasympathetic nervous systems, resulting in autonomic dysfunction that alters the electrical stability of the atria. Inflammatory cascades activated by infections and inflammation, along with oxidative stress, further contribute to atrial remodeling and the development of AF. The relationship between infections, inflammation, and AF is complex, and ongoing research aims to better understand these mechanisms for potential therapeutic interventions [15, 16].

The precise underlying mechanism of NOAF in COVID-19 remains incompletely elucidated, although a number of presumed mechanisms have been postulated. These include COVID-19-triggered systemic inflammation, viral myocarditis, dysregulation of the autonomic nervous system, hypercoagulability and thromboembolism, as well as electrolyte homeostasis imbalance and cardiac injury [17, 18].

A systematic review and meta-analysis of 280,589 community acquired pneumonia (CAP) patients found a prevalence of new-onset AF up to 7.6%, with potential rates of up to 13% [19]. In one of the reviews, the average occurrence of new-onset atrial fibrillation (AF) in patients with sepsis was found to be 20.6%. This incidence varied between retrospective studies (14.7%) and prospective studies (31.6%) [20]. However, another study of critically ill patients with sepsis, severe sepsis, and septic shock, the weighted mean incidence of AF was determined to be 8% (with a range of 0% to 14%), 10% (with a range of 4% to 23%), and 23% (with a range of 6% to 46%), respectively [21].

The frequency of new onset atrial fibrillation in COVID 19 patients have not been studied in our population. As absolute treatment regimen to combat this deadly virus is still under investigation and the better health facility is not in reach of every average person in resource limited settings due to economic instability, so recognition of new on-set atrial fibrillation and early intervention is important to reduce the associated morbidity and mortality and providing patient care. We carried out this study to help clinicians understand the potential damage to the cardiovascular system caused by COVID-19 and strengthen the monitoring and preservation of cardiac function.

## Materials and methods

### Study design

A retrospective cross-sectional that included all consecutive patients admitted to Aga Khan University Hospital in Karachi, a tertiary care facility. The study population consisted of patients who were either confirmed to have COVID-19 or tested negative for the virus and were admitted to the hospital from 1^st^ June 2021 to 30^th^ December 2021.

The study received approval from the Ethical Review Committee (ERC) of the Aga Khan University Hospital Institutional Review Board, with a waiver of informed consent.

All cases of COVID‐19 were confirmed through real‐time reverse‐transcriptase polymerase chain reaction assays on nasopharyngeal swabs. Data was manually extracted from the electronic health record of AKU on Excel sheet.

### Eligibility criteria

The study population consisted of adult patients, both male and female, who were hospitalized at the time of inclusion. Inclusion criteria were limited to patients above the age of 18 years. However, patients with a history of atrial fibrillation or those who were already receiving rate or rhythm control medications for atrial fibrillation were excluded to maintain the homogeneity of the study population and reduce confounding factors.

### Data collection

The demographics (age, sex), diabetes mellitus (DM), hypertension (HTN), Non-ST-elevation myocardial infarction (NSTEMI), ischemic heart disease (IHD), cerebrovascular accident (CVA), chronic Kidney disease (CKD), acute kidney injury (AKI), obstructive lung disease (OLD), pneumonia (PNA), cytokine release syndrome (CRS), acute respiratory distress syndrome (ARDS), New onset atrial fibrillation (NOAF) were abstracted on Excel sheet from the medical health records.

All standard 12‐lead electrocardiograms (ECGs) recorded during hospitalization were reviewed. ECGs were recorded at 25 mm/s and 1 mV/cm according to standard protocol. Use of antiviral medications, steroids, and vasopressors during hospitalization were also abstracted. Cytokine release syndrome was defined as a severely over-reactive immune system that progresses in an unregulated manner which was characterized by increase in inflammatory markers particularly CRP and Ferritin.

### Statistical analysis

Data were analyzed on SPSS software Version 26.0. Descriptive statistics were used to analyze the baseline characteristics of the patients, with the results reported as frequencies and percentages. Univariate and multiple-adjusted regression analysis were performed to evaluate the relationship between variables and the outcome. Multivariable logistic regression, adjusted for demographics, pre-existing medical conditions, acute kidney injury, pneumonia, cytokine release syndrome, acute respiratory distress syndrome was performed to assess the predictors of the outcome (new onset atrial fibrillation). Unadjusted odd ratio (UOR) and adjusted odd ratios (AORs) were presented with a 95% confidence interval and a p-value of < 0.05 was considered statistically significant.

## Results

In the time frame from June to December 2021, 1,313 patients who met the inclusion criteria of our study were enrolled as participants. These patients were then stratified into two groups based on COVID-19 status: the study group consisted of 626 patients who tested positive for COVID-19 (47.7%) and the control group consisted of 687 patients who tested negative for COVID-19 (52.3%). The mean age of the participants was found to be 58.98 years, with a standard deviation (SD) of 17.01. Both groups showed a male dominance, with 360 (57.5%) patients in the study group and 353 (51.4%) patients in the control group, while 327 (42.5%) patients in the study group and 273 (48.6%) patients in the control group identified as female. In our cohort, 518 (82.7%) of the COVID-19 positive patients were admitted to the general medicine ward, while 108 (17.2%) were admitted to the ICU. In terms of severity of COVID positive patients, 139 (22.2%) individuals had non-severe COVID pneumonia, 342 (54.6%) had severe pneumonia, and 144 (23%) had critical COVID-19 pneumonia. On the other hand, the Covid-19 negative patient 590 (85%) were in ward and 97 (14.1%) were admitted to ICU.

The study group also showed a higher incidence of new-onset atrial fibrillation, with 85 (13.6%) patients in the COVID-19 positive group developing the condition compared to 43 (5.2%) patients in the COVID-19 negative group. Additionally, the mortality rate was higher in the COVID-19 positive group, with 78 (12.5%) compared to 36 (5.2%) patients in the control group during their hospital stay. The details of the two groups are presented in Table 1.

**Table 1:** Characteristics of the two groups.

Variables	Covid Positive (n = 626)	Covid Negative (n = 687)
Age(years) Mean ± SD	58 ± 15	59 ±19
Sex (male)	360 (57.5%)	353 (51.4%)
DM	38 (6.1%)	264 (38.4%)
HTN	29(4.6%)	318 (46.3%)
NSTEMI	31 (5%)	63 (9.2%)
IHD	82 (13.1%)	84(12.2%)
CVA	16(2.6%)	16 (2.3)
CKD	33 (5.3%)	54 (7.7%)
AKI	82 (13.1%)	165 (23.7%)
Obstructive lung diseases	8 (1.3%)	16 (1.9%)
Pneumonia	56 (8.9%)	58 (8.4%)
Cytokine release syndrome	90 (14.4%)	0 (0%)
ARDS	18 (2.9%)	0 (0%)
Dyslipidemia	14 (2.2%)	70 (9.8%)
NOAF	85 (13.6%)	43(6.3%)
Death	78 (12.5%)	36 (5.2%)

Diabetes mellitus (DM), Hypertension (HTN), Non-ST-elevation myocardial infarction (NSTEMI), Ischemic heart disease (IHD), Cerebrovascular accident (CVA), Chronic Kidney disease (CKD), Acute kidney injury (AKI), obstructive lung disease (OLD), Pneumonia (PNA), Cytokine release syndrome (CRS), Acute respiratory distress syndrome (ARDS), New onset atrial fibrillation (NOAF).

The unadjusted model, which investigated NOAF as an outcome, revealed an association between age, NSTEMI, IHD, CVA, and COVID (P-value < 0.05) and the occurrence of new-onset atrial fibrillation. After running a logistic regression and obtaining the adjusted model, which controlled for other demographics and comorbidities while examining new-onset atrial fibrillation as the outcome, the association between COVID and new-onset atrial fibrillation was stronger (AOR 3.86 [95% CI, 2.31–6.44], p-value < 0.01), compared to the unadjusted model (UOR 2.35 [95% CI, 1.60–3.45], p value < 0.01). In the adjusted model, the association between AKI, IHD and CVA and NOAF was no longer statistically significant. Only age, NSTEMI, Pneumonia (PNA), and COVID showed a significant association (p-value < 0.05) with new-onset atrial fibrillation. The details can be found in Table 2.

**Table 2:** Unadjusted odd ratio (UOR) and adjusted odds ratio (AOR) (95% CI), NOAF as an outcome.

Variables	UOR (95% CI)	P-value	AOR (95% CI)	P-value
Age (years)	1.03(1.018–1.0450)	< 0.01	1.02(1.021–1.042)	< 0.01
Sex (Male)	1.13 (0.75–1.71.)	0.536	0.99 (0.64–1.53)	0.97
DM	1.07 (0.70–1.65)	0.730	1.55 (0.84–2.86)	0.160
HTN	1.10 (0.73–1.65)	0.647	1.39 (0.74–2.63)	0.303
NSTEMI	2.99 (1.77–5.05)	<0.01	2.40 (1.32–4.38)	0.004
IHD	2.01 (1.27–3.18)	0.003	1.52 (0.91–2.53)	0.104
CVA	2.68 (1.13–6.33)	0.024	2.07 (0.77–5.61)	0.149
CKD	1.72 (0.92–3.20)	0.086	1.14 (0.57–2.26)	0.706
AKI	1.82 (1.20–2.76)	0.004	1.49 (0.92–2.42)	0.101
Obstructive lung diseases	0.45 (0.06–3.44)	0.449	0.43 (0.05–3.37)	0.422
Pneumonia	3.79 (2.37–6.05)	< 0.01	3.12 (1.85–5.25)	< 0.01
Cytokine release syndrome	1.30 (0.69–2.45)	0.409	0.86 (0.42–1.75)	0.691
ARDS	2.69 (0.87–8.32)	0.084	1.44 (0.38–5.40)	0.587
Dyslipidemia	0.46 (0.16–1.29)	0.141	0.37 (0.12–1.16)	0.089
Covid-19	2.35 (1.60–3.45)	< 0.01	3.86 (2.31–6.44)	< 0.01

Unadjusted odd ratio (UOR), adjusted odd ratio (AOR), Diabetes mellitus (DM), Hypertension (HTN), Non-ST-elevation myocardial infarction (NSTEMI), Ischemic heart disease (IHD), Cerebrovascular accident (CVA), Chronic Kidney disease (CKD), Acute kidney injury (AKI), obstructive lung disease (OLD), Pneumonia (PNA), Cytokine release syndrome (CRS), Acute respiratory distress syndrome (ARDS), New onset atrial fibrillation (NOAF).

A secondary analysis was conducted to examine the association between new-onset atrial fibrillation and death as the outcome of interest. The unadjusted analysis revealed that several factors, including age, NSTEMI, IHD, obstructive lung disease, pneumonia, Cytokine release syndrome, Acute respiratory distress syndrome, and COVID-19, were significantly associated with death (p<0.05). However, NOAF was not found to have a significant association with death (UOR 1.09 [95% CI, 0.58–2.05], p-value 0.770). After conducting a logistical regression to adjust for the effect of other demographics and comorbidities, NOAF remained not significantly associated with death (AOR 0.52 [95% CI, 0.26–1.07], p-value 0.078). In the adjusted model, only age, hypertension, obstructive lung diseases, pneumonia, cytokine release syndrome, ARDS, and COVID-19 remained statistically significant predictors of death (p-value < 0.05), and the association between NSTEMI and IHD with death was no longer significant. The detailed is depicted in Table 3.

**Table 3:** Unadjusted odds ratio (UOR) and adjusted odds ratio (AOR) (95% CI), death as an outcome.

Variables	UOR (95% CI)	P-value	AOR (95% CI)	P-value
Age (years)	1.03 (1.02–1.05)	<0.001	1.03 (1.02–1.05)	<0.001
Sex (male)	1.3 (0.80–2.1)	0.27	1.09 (0.65–1.82)	0.74
DM	1.09 (0.70–1.71)	0.679	1.27 (0.64–2.50)	0.483
HTN	1.37 (0.91–2.08)	0.128	2.83 (1.41–5.70)	0.003
NSTEMI	2.14 (1.19–3.87)	0.011	1.76 (0.87–3.56)	0.113
IHD	1.98 (1.22–3.21)	0.005	1.55 (0.91–2.64)	0.105
CVA	1.52 (0.52–4.41)	0.441	1.22 (0.37–4.02)	0.178
CKD	1.78 (0.94–3.40)	0.076	1.64 (0.79–3.39)	0.706
AKI	1.11 (0.68–1.79)	0.664	0.80 (0.45–1.42)	0.453
Obstructive lung diseases	3.39 (1.21–9.43)	0.019	3.76 (1.27–11.1)	0.017
Pneumonia	3.50 (2.13–5.73)	< 0.01	3.46 (1.94–6.19)	< 0.01
Cytokine release syndrome	4.55 (2.77–7.49)	< 0.01	4.13 (2.28–7.45)	< 0.01
ARDS	18.13 (6.90–47.9)	< 0.01	9.39 (3.05–28.9)	< 0.01
Dyslipidemia	0.99 (0.16–1.29)	0.989	0.71 (0.27–1.85)	0.491
Covid-19	2.57 (1.70–3.88)	< 0.01	3.92 (2.12–7.22)	< 0.01
NOAF	1.09 (0.58–2.05)	0.770	0.52 (0.26–1.07)	0.078

Unadjusted odd ratio (UOR), adjusted odd ratio (AOR), Diabetes mellitus (DM), Hypertension (HTN), Non-ST-elevation myocardial infarction (NSTEMI), Ischemic heart disease (IHD), Cerebrovascular accident (CVA), Chronic Kidney disease (CKD), Acute kidney injury (AKI), obstructive lung disease (OLD), Pneumonia (PNA), Cytokine release syndrome (CRS), Acute respiratory distress syndrome (ARDS), New onset atrial fibrillation (NOAF).

## Discussion

This study was conducted within a single tertiary care center located in a lower middle income country and was designed as a single center-based study. The study population consisted of 626 patients diagnosed with COVID-19 and was analyzed to determine the incidence and association of new onset atrial fibrillation in COVID-19 hospitalized patients and its impact on survival.

The results of our study found the prevalence of new onset atrial fibrillation to be 13.6%. This incidence rate is higher compared to the findings of a recent multi-center study conducted in the United States, which included 27,851 patients and concluded that the incidence rate of new-onset atrial fibrillation among COVID-19 patients was 5.4% [14]. An earlier systematic review had placed the incidence rates of NOAF to be in between 4.33% and 14.61% [10]. These findings suggest that the incidence of new-onset atrial fibrillation in COVID-19 patients may be influenced by healthcare system factors such as variations in clinical awareness, screening protocols, or access to healthcare resources, may influence the identification and diagnosis of atrial fibrillation cases. Disparities in healthcare settings and practices across different regions or centers could contribute to differences in the reported prevalence rates. The rate of incidence of atrial fibrillation in the COVID negative group was 6.3%, less than half of that in COVID positive patients. The higher prevalence of NOAF in COVID-19 positive patients (13.6%) compared to COVID-negative patients (6.3%) may be explained by COVID-19-related factors such as systemic inflammation, direct viral involvement in cardiac tissue, and the exacerbation of pre-existing risk factors. Additionally, the systemic effects of COVID-19, including hypercoagulability and thromboembolic events, could contribute to atrial fibrillation development.

Atrial fibrillation is the most prevalent supraventricular arrhythmia observed in COVID-19 patients and is associated with an increased risk of complications. Timely recognition and treatment of AF are crucial, with anticoagulation therapy being a cornerstone in preventing thrombus formation and dislodgment. COVID-19 patients with AF are particularly vulnerable to adverse outcomes due to the underlying inflammatory response, endothelial dysfunction, and hypercoagulable state. Anticoagulants play a vital role in managing AF by inhibiting clotting and reducing the risk of atrial thrombus formation [22]. Early initiation of anticoagulation aims to mitigate the potential complications associated with thromboembolism, such as stroke and systemic embolism [13]. However, treatment decisions should consider individual patient factors and involve a multidisciplinary approach to optimize care for COVID-19 patients with AF.

The development of new-onset atrial fibrillation was found to be associated with older age and comorbidities such as NSTEMI and pneumonia. Both univariate and multiple-adjusted regression analysis revealed that patients with COVID had a higher likelihood of experiencing NOAF. Wollborn J, et al. reported COVID-19 patients to have 1.19 times more risk of developing atrial fibrillation than those COVID negative [23]. In a prospective cohort study of 280,592 elderly patients in the US, it was found that COVID-19 status exhibited a stronger independent association with AF compared to traditional cardiovascular co-morbidities such as congestive heart failure and coronary artery disease [24].

While it is evident from existing body of literature that an association exists between NOAF and mortality in COVID-19 patients; however, no association was found in our study this can be due to inappropriate study design, potentially inadequate sample size, and uncertainty regarding the reliability of assessing NOAF and mortality. Nevertheless, the presence of COVID was identified as a significant risk factor for mortality. The individuals who died during their treatment were more likely elderly, suffering from hypertension, obstructive lung disease, pneumonia, cytokine release syndrome, acute respiratory distress syndrome. Rosenblatt AG, et al. conducted a multi-center study with 30,999 participants but failed to establish a causal association between atrial fibrillation and mortality of patients [14]. However, other studies conducted have shown a significant association between atrial fibrillation and mortality [10]. Szarpak, et al. reports a 1.8 times increased mortality in COVID patients with NOAF than in COVID patients without any new-onset atrial fibrillation [18]. Although not fully understood, the association between mortality and new-onset atrial fibrillation is widely regarded, the possible pathophysiology behind which the mechanical stress on the cardiomyocytes (atrial remodeling), the hyperinflammatory condition (cytokine storm and oxidative stress), genetic susceptibility, and the electrical instability of atrial arrhythmias, etc. [25]. Romiti, et al. performed a systematic review and meta-analysis of 14 studies, they concluded that there was four times higher risk of mortality in COVID positive patients with atrial fibrillation as opposed to COVID positive patients with no atrial fibrillation [12].

The association between hypertension and mortality in the COVID-positive group, specifically in patients with new-onset atrial fibrillation, can be attributed to the combined effects of increased thromboembolic risk associated with both conditions. Atrial fibrillation and hypertension contribute to a prothrombotic state, leading to the formation and dislodgment of blood clots. COVID-19 infection exacerbates this risk by inducing systemic inflammation and endothelial dysfunction, further promoting clot formation [26]. These factors likely contribute to the adverse outcomes observed in COVID-positive patients with atrial fibrillation and hypertension. However, a comprehensive understanding of the underlying mechanisms necessitates further investigation.

In the future, healthcare providers should be aware and attentive to the potential cardiac impacts of COVID-19 on their patients. It is important for them to closely monitor patients for any signs of cardiac complications, including the onset of AF, and to provide timely and appropriate care to mitigate the potential negative impact on the patients’ health and survival.

### Limitations

This study has some limitations that should be considered when interpreting the results. One such limitation is the retrospective design of the cohort, which has the potential to introduce bias. A prospective cohort study design would be a more ideal method for this analysis. Additionally, the atrial fibrillation was recorded via ECG, which might have missed some AF cases, and using a Holter monitor could have provided more comprehensive AF detection. The sample population was not stratified based on the severity of their disease, and there is no follow-up data or information on patient outcomes. Furthermore, the observational design of the study may not have fully accounted for all confounders, and the treatment regimen for atrial fibrillation is not specified or described.

## Conclusion

The study aimed to uncover the association and incidence of new onset atrial fibrillation among COVID-19 hospitalized patients. The results of the study suggest that COVID-19 increases the risk of developing new onset atrial fibrillation, which is a potentially serious and life-threatening complication. The findings highlight the need for close monitoring of cardiovascular complications in COVID-19 patients, particularly in those who are at increased risk of atrial fibrillation due to other underlying conditions. The study also underscores the importance of early recognition and management of new onset atrial fibrillation in COVID-19 patients, which may help to prevent serious complications and improve outcomes. These results contribute to our understanding of the complex relationship between COVID-19 and cardiovascular health and may inform future research and clinical practice in this area.

## Supporting information

S1 Data (XLSX)
